# Sotetsuflavone Induces Autophagy in Non-Small Cell Lung Cancer Through Blocking PI3K/Akt/mTOR Signaling Pathway *in Vivo* and *in Vitro*

**DOI:** 10.3389/fphar.2019.01460

**Published:** 2019-12-05

**Authors:** Shaohui Wang, Xiaoling Xu, Yanlan Hu, Tao Lei, Tongxiang Liu

**Affiliations:** ^1^Key Laboratory of Ethnomedicine (Minzu University of China), Minority of Education, Beijing, China; ^2^Medical College, Qingdao Binhai University, Qingdao, China; ^3^School of Pharmacy, Minzu University of China, Beijing, China; ^4^Department of Medical Oncology, Institute of Cancer Research and Basic Medical Sciences of Chinese Academy of Sciences, Cancer Hospital of University of Chinese Academy of Sciences, Zhejiang Cancer Hospital, Hangzhou, China

**Keywords:** sotetsuflavone, non-small cell lung cancer, apoptosis, autophagy, PI3K/Akt/mTOR signaling pathway

## Abstract

Non-small cell lung cancer (NSCLC) is a globally scaled disease with a high incidence and high associated mortality rate. Autophagy is one of the important physiological activities that helps to control cell survival, influences the dynamics of cell death, and which plays a crucial role in the pathophysiology of NSCLC. Sotetsuflavone is a naturally derived and occurring flavonoid, and previous studies have demonstrated that sotetsuflavone possesses potential anti-cancer activities. However, whether or not sotetsuflavone induces autophagy, as well as has effects and influences cell death in NSCLC cells remains unclear. Thus, in our study, we examined and elucidated the roles and underlying mechanisms of sotetsuflavone upon the dynamics of autophagy in NSCLC *in vivo* and *in vitro*. The results indicated that sotetsuflavone was able to inhibit proliferation, migration, and invasion of NSCLC cells. Mechanistically, sotetsuflavone was able to induce apoptosis by increasing the levels of expression of cytochrome C, cleaved-caspase 3, cleaved-caspase 9, and Bax, and contrastingly decreased levels of expression of Bcl-2. In addition, we also found that decreased levels of expression of cyclin D1 and CDK4 caused arrest of the G0/G1 phases of the cell cycle. Furthermore, we also found that sotetsuflavone could induce autophagy which in turn can play a cytoprotective effect on apoptosis in NSCLC. Sotetsuflavone-induced autophagy appeared related to the blocking of the PI3K/Akt/mTOR pathway. Our *in vivo* study demonstrated that sotetsuflavone significantly inhibited the growth of xenograft model inoculated A549 tumor with high a degree of safety. Taken together, these findings suggest that sotetsuflavone induces autophagy in NSCLC cells through its effects upon blocking of the PI3K/Akt/mTOR signaling pathways. Our study may provide a theoretical basis for future clinical applications of sotetsuflavone and its use as a chemotherapeutic agent for treatment of NSCLC.

## Introduction

Globally, lung cancer is the leading cause of cancer-related deaths and the most commonly diagnosed type of cancer, and continues to threaten human health and quality of life (Bui et al., 2018). In 2012, there were 1.8 million newly diagnosed cases of lung cancer, accounting for 12.9% of all newly diagnosed cancer cases of any type, and there were 1.6 million deaths from this disease accounting for 19.4% of all cancer deaths. (Ferlay et al., 2015). Non-small cell lung cancer (NSCLC) accounts for 85% of all types of lung cancer diagnoses. Adenocarcinoma is the most common histological subtype of NSCLC, accounting for nearly 40% of diagnoses, and it is still the leading cause of cancer death (Dela Cruz et al., 2011; Torre et al., 2015). In contrast to the steady increase in rates of survival in most other types of cancers as treatment options and outcomes have improved, advances in treatments for types of lung cancers have been relatively fewer in comparison, and the 5-year relative survival rate is only currently 18% (Siegel et al., 2017; Zhang et al., 2018b). Traditional Chinese medicines have shown potential anticancer effects, and might be used as new sources of anticancer drugs and neoadjuvant chemotherapy treatments, not only to improve the efficacy of and to reduce the side effects of chemotherapy. (Yang et al., 2010; Xu et al., 2015). However, there is currently an absence of scientific basis for the use and application of these Chinese medicines for treatment of cancers (Yang et al., 2010), therefore, identifying and finding safe and effective drugs such as may be derived from traditional Chinese medicines is the part of the key requirements needed to achieve better therapeutic outcomes and ideal patient prognoses.

Flavonoids are the most common and abundant polyphenols humans are typically exposed to in the course of normal daily life and have a wide range of pharmacological effects (Fu et al., 2016). In vitro studies have shown that anti-cancer effects of flavonoids may be related to their induction of inhibition of: cell proliferation, cell adhesion, cell invasion, cell differentiation, cell cycle phases, and cell apoptosis (Yan et al., 2017; Zhang et al., 2018a). In vivo studies have shown that flavonoids were able to inhibit carcinogenesis, and the effect was mainly characterized as being related to molecular events during the stages of initiation, promotion, and progression (Zhao et al., 2017). Clinical trials that have examined the use of flavonoids have been conducted with the goal of achieving cancer prevention or in order to obtain better therapeutic effect (Awan et al., 2016; Zhang et al., 2018a). Therefore, flavonoids can and should be further developed as potentially promising compounds for chemoprevention and chemotherapy of cancer (Zhang et al., 2018a). Flavonoids including such as quercetin (Psahoulia et al., 2007), baicalein (Aryal et al., 2014), and genistein (Prietsch et al., 2014) have been shown to be able to induce autophagy-related death in certain and specific types of tumor cells. Studies have shown that autophagy is complicated in that it can act to both promote and inhibit tumor growth and that autophagy also develops and is carried out differentially under different experimental environments (Aryal et al., 2014). Autophagy is a biological process in which cytokines and unfolded proteins in cells need to be degraded and metabolized within lysosomes (Gozuacik and Kimchi, 2004). The dynamics of autophagy also can play a significant role in the development and progression of cancers and is considered to be an important mechanism of cell death (Gozuacik and Kimchi, 2004; Wang et al., 2017). In many types of malignant tumors, PI3K/AKT is often in an abnormal state of activation which has been characterized as related to the occurrence and development of various types of cancers (Bakin et al., 2000). Moreover, it is reported that the PI3K/Akt/mTOR signaling pathway plays a significant role in effecting the dynamics of cell autophagy and has become increasingly researched for use as development to target tumors (Porta et al., 2014; Wang et al., 2017). Previous research has indicated that sotetsuflavone possesses potential anti-cancer activities (Wang et al., 2018b). However, whether or not sotetsuflavone can induce autophagy in NSCLC cells is unknown and the role of sotesuflavone in cell death in NSCLC cells is unclear. Thus, in our study, we explored the effects and underlying mechanisms of sotetsuflavone upon the dynamics of autophagy in NSCLC cells both *in vivo* and *in vitro*.

## Results

### Sotetsuflavone Inhibits Proliferation of NSCLC Cells

**Figure 1:**
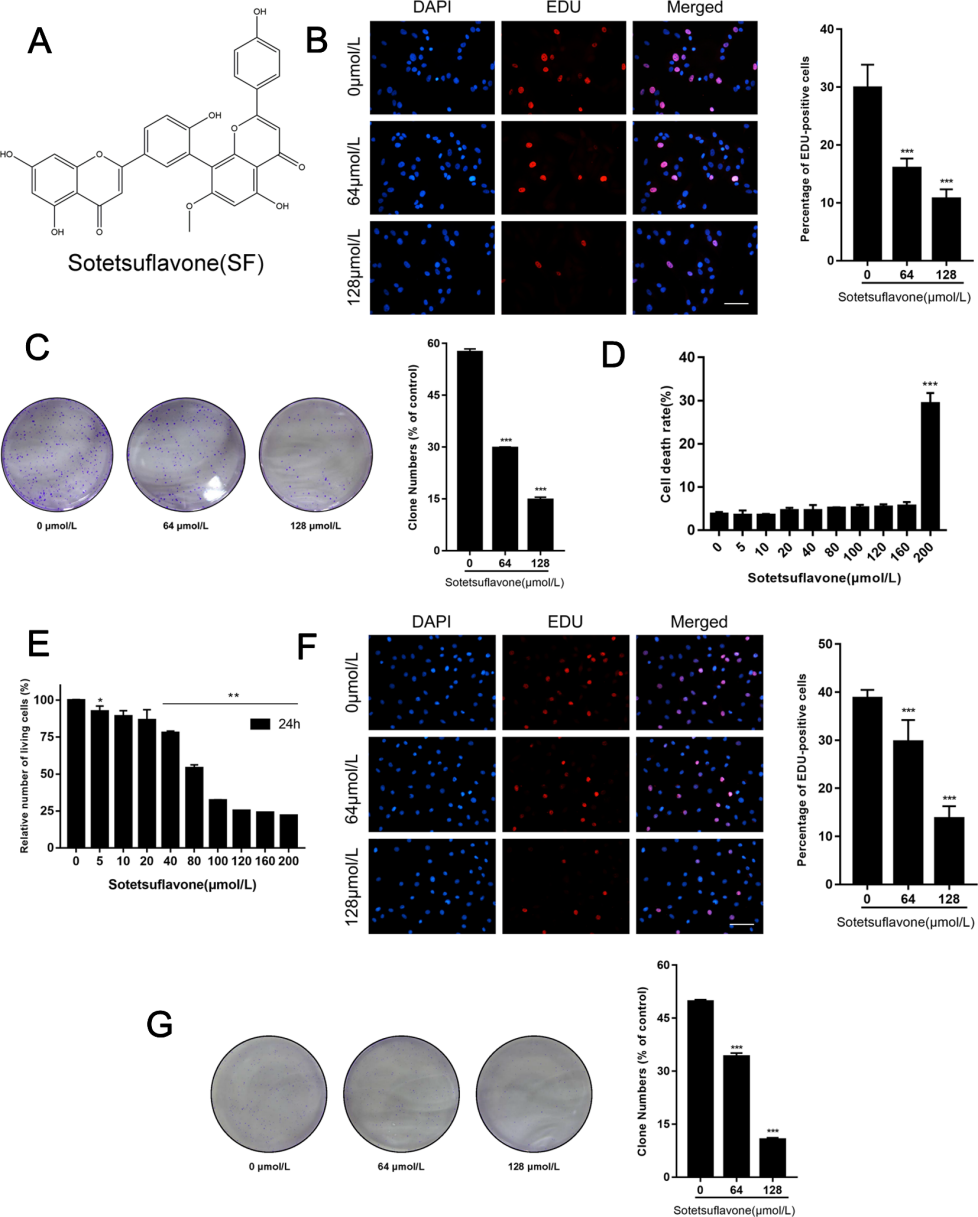
Sotetsuflavone inhibits proliferation of non-small cell lung cancer cells. **(A)** The chemical structure of sotetsuflavone with a molecular weight of 552.491 g/mol. Sotetsuflavone is a biflavonoid which is formed by aapegenin linked by the 8th position of the A ring to the 3′ position of the 7-methylapigenin B ring. **(B)** Proliferating A549 cells were labeled with EDU (red), cell nuclei were stained with DAPI (blue), and the percentage of EDU-positive A549 cells was quantified. Original magnification, ×200. (**p < 0.001 vs. control). **(C)** Results from A549 cell colony formation assays (***p < 0.001 vs. control). **(D)** The toxicity of sotetsuflavone on normal lung epithelial cells (BEAS-2B) was detected by use of trypan blue staining. Living cell rate = total number of living cells/(total number of living cells + total number of dead cells) × 100% (***p < 0.001 vs. control). **(E)** The relative number of H1650 living cells treated with different concentrations of sotetsuflavone for 24 h (*p < 0.05, **p < 0.01 vs. control). **(F)** Proliferating H1650 cells were labeled with EDU (red), cell nuclei were stained with DAPI (blue), and the percentage of EDU-positive H1650 cells was quantified. Original magnification, ×200 (***p < 0.001 vs. control). **(G)** Colony formation assays were also performed to measure the growth of H1650 cells (***p < 0.001 vs. control).

The structure of sotetsuflavone is shown in **Figure 1**. In the early stages of analysis, we used the 3-(4, 5-dimethylthiazol-2-yl)-2, 5-diphenyltetrazolium bromide (MTT) method to detect the function and effects of sotetsuflavone on the viability of A549 cells. Results indicated that sotetsuflavone had a strong inhibitory effect on the levels of activity of A549 cells (Wang et al., 2018a). To further verify whether or not sotetsuflavone is a potent inhibitor of A549 cell proliferation, we used 5-ethynyl-20-deoxyuridine (EDU) and colony formation experiments. Compared to the control group, the percentage of EDU that was incorporated into living cells for the sotetsuflavone treatment group was significantly reduced (**Figure 1**). In addition, the results from experimental colony formation assays indicated that sotetsuflavone was able to significantly inhibit the proliferation of A549 cells (**Figure 1**). We also confirmed whether or not sotetsuflavone had any toxic effects on normal human lung epithelial cells (BEAS-2B). As demonstrated in **Figure 1**, sotetsuflavone (0–160 µmol/L) had no obvious toxic effects on BEAS-2B cells, while sotetsuflavone (200 µmol/L) had a significantly increased level of cytotoxicity when applied to BEAS-2B cells. We further verified this result using H1650 cells. After 24 h of treatment with different doses sotetsuflavone (0, 5, 10, 20, 40, 80, 100, 120, 160, and 200 µmol/L), MTT assays were used to detect the effect of sotetsuflavone on the inhibition of growth of H1650 cells (**Figure 1**). The inhibition rate was 0%, 7.6%, 10.9%, 13.5%, 22.1%, 45.9%, 67.9%, 74.8%, 76.2%, and 78.2%, and the IC_50_ value was 67.54 µmol/L. Both EDU and colony formation experiments also showed similar results (**Figures 1F, G**). The above results indicated that sotetsuflavone was able to inhibit the growth of H1650 cells in a both a dose- and time-dependent manner. These results further indicated that sotetsuflavone was able to inhibit the proliferation of NSCLC cells. In summary, we adjusted the following experimental concentrations to 0 (control), 64, and 128 µmol/L.

### Sotetsuflavone Inhibits the Migration and Invasion, and Induces Apoptosis and Cell Cycle Arrest in NSCLC Cells

**Figure 2:**
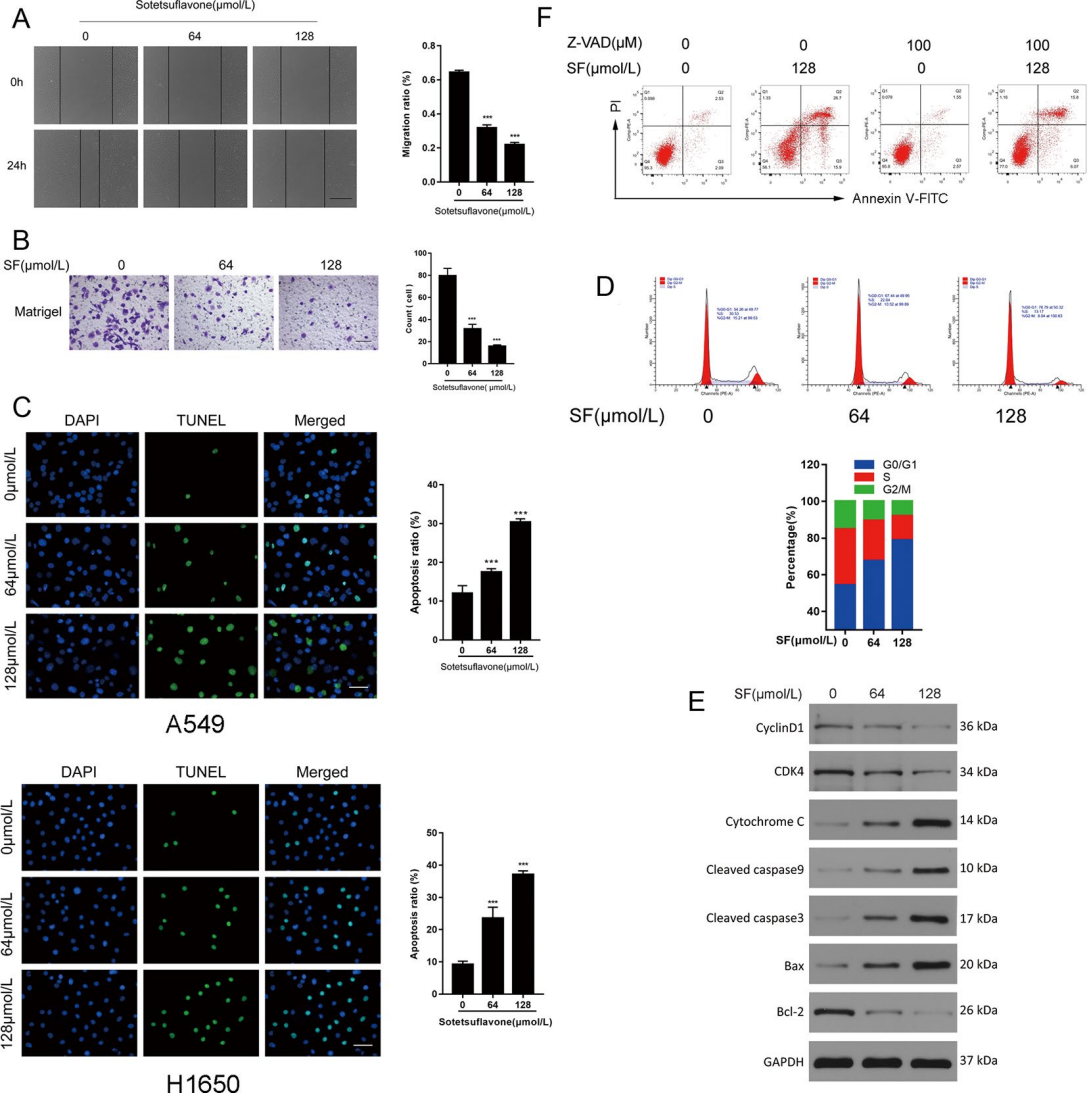
Sotetsuflavone inhibits the migration and invasion, and induces apoptosis and cell cycle arrest in non-small cell lung cancer cells. **(A)** H1650 cells were treated with sotetsuflavone for 24 h, and the cell scratch assay was performed to evaluate the migration ability of H1650 cells. Original magnification×40 (***p < 0.001 vs. control). **(B)** Transwell invasion assays were used to evaluate the effect of sotetsuflavone on the invasion ability of H1650 cells. Original magnification×100 (***p < 0.001 vs. control). **(C)** TUNEL apoptosis assay in A549 and H1650 cells. Apoptotic nuclei were labeled with TUNEL (green), and DNA was stained by DAPI (blue). Original magnification×200 (***p < 0.001 vs. control). **(D)** H1650 cells were treated with sotetsuflavone for 24 hours and cell cycle phases were detected by flow cytometry. **(E)** Western blotting analysis of Cyclin D1, CD4, Bax, Bcl-2, cleaved-caspase 3, cleaved-caspase 9, and cytochrome C in H1650 cells. **(F)** Flow cytometric analysis of Annexin V-FITC/PI staining in H1650 cells treated with or without sotetsuflavone (128 μM) in combination with Z-AVD (100 μM) for 24 h.

Previously, we demonstrated that sotetsuflavone was able to inhibit the migration and invasion, and able to induce apoptosis and cycle arrest of A549 cells (Wang et al., 2018a; Wang et al., 2018b). Thus, we used Cell scratch assays, Transwell invasion assays, Tunel assays, and flow cytometry to test whether or not sotetsuflavone was able to inhibit the migration and invasion, as well as induce apoptosis and cell cycle arrest in H1650 cells. Coincidently, the application of sotetsuflavone had a significant dose-dependent effect upon inhibiting H1650 cell migration and invasion (**Figures 2A, B**), and inducing both H1650 cell apoptosis and cell cycle arrest (**Figures 2C, D**). We further examined the levels of expression of cycle-related proteins and apoptosis-related proteins through WB assays. The results from WB assays indicated that cyclin D1, CD4, and Bcl-2 proteins were downregulated, whereas the levels of expression of Bax, cleaved-caspase 3, cleaved-caspase 9, and cytochrome C were upregulated (**Figure 2E**). Furthermore, in order to investigate the importance of caspase activation in cell apoptosis induced by sotetsuflavone, we applied a pretreatment of H1650 with Z-VAD (a Pan-caspase inhibitor) in order to block caspase. As shown in **Figure 2F**, the application of Z-VAD significantly reduced the effect of sotetsuflavone-induced cell death. These results fully demonstrate that sotetsuflavone was able to inhibit the migration and invasion as well as induce apoptosis and cycle arrest of NSCLC cells. Interestingly, apoptosis that was induced by the application of sotetsuflavone was mainly dependent upon caspase activation.

### Sotetsuflavone Induces Autophagy in NSCLC A549 Cells

**Figure 3:**
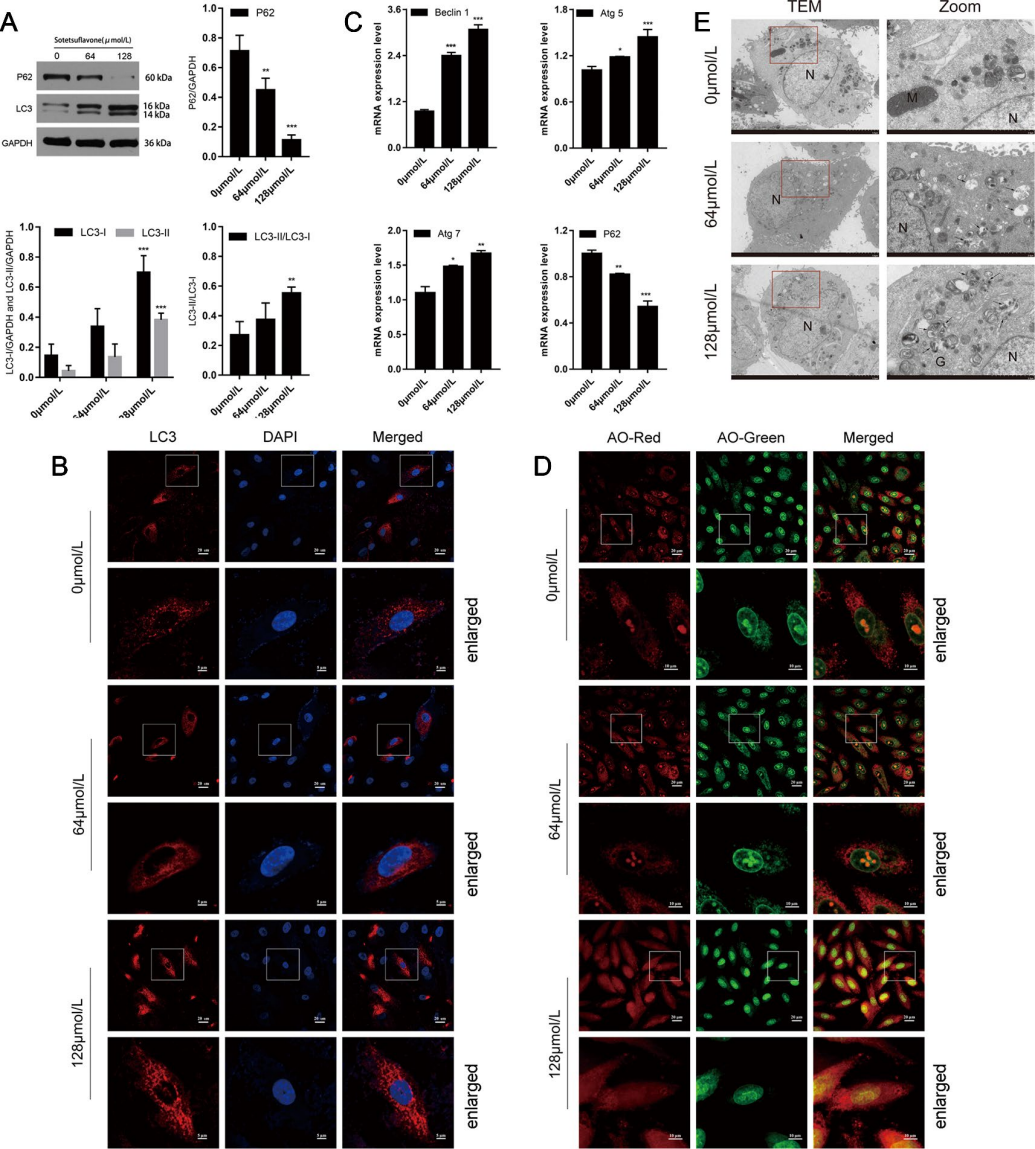
Sotetsuflavone induces autophagy in non-small cell lung cancer cells. **(A)** The levels of expression of autophagy-related protein (LC3B-I/LC3B-II, P62) in A549 cells treated with different concentrations of sotetsuflavone for 24 h was detected by Western blotting analysis. GAPDH was used as a loading control (**p < 0.01, ***p < 0.001 vs. control). **(B)** A549 cells were treated with or without sotetsuflavone for 24 h, and LC3 spots were observed under a confocal microscope. Scale bar: 5 μm. **(C)** RT-PCR was used to detect the levels of expression of autophagy-related genes beclin1, Atg5, Atg7, and P62. Data are expressed as the mean ± SD (*p < 0.05, **p < 0.01, ***p < 0.001). **(D)** A549 cells were treated in the absence or presence of sotetsuflavone for 24 h, and then stained with acridine orange (AO). The corresponding merged images are shown on the right. **(E)** TEM image which depicts the ultrastructure of A549 cells treated without or with sotetsuflavone (64 and 128 μmol/L) for 24 h. N, Nucleus; M, Mitochondria; G, Golgi apparatus. Black arrows indicate autophagosomes or autophagic lysosomes. Scale bar: 2 μm.

Next, we examined whether or not sotetsuflavone was able to induce autophagy in NSCLC A549 cells. Firstly, examined the degree of transformation of LC3-I into lipidizing LC3-II. LC3-II is a well-known and classical marker of autophagosome formation, and an increase of LC3-II would represent the initiation of autophagy (Yang and Klionsky, 2010; Panda et al., 2015). Meanwhile, we also examined and detected the levels of expression of P62, and since P62 can be degraded by autophagy, we therefore used the measure of P62 in order to reflect the strength of autophagy. When LC3-II was increased, and P62 was decreased, it indicated that autophagy was fluent. When LC3-II was increased and P62 also increased, it indicated that the initiation of autophagy was normal, but downstream examinations of autophagy are unreasonable, and the phagosomes and lysosomes cannot fuse (Panda et al., 2015). Our results thus indicated that sotetsuflavone was able to increase the levels of autophagy of A549 cells by enhancing LC3-II conversion and induced the decreased the expression of P62 (**Figure 3**). To confirm the evidence suggesting that autophagy formation was induced by sotetsuflavone, we next detected the distribution of LC3 spots. The results of immunofluorescence tests revealed that when compared with results for the control group that the endogenous LC3 spots were significantly increased in the sotetsuflavone treatment group (**Figure 3**). Autophagy flux is a common and widely used measure to help define the process of dynamic autophagy, thus, we observed the changes of levels of expression of LC3 after A549 cells were treated with or without autophagy inhibitors (CQ). As shown in **Figure S1**, the applied combination of both sotetsuflavone and CQ resulted in the accumulation of LC3. In addition, we studied the levels of expression of autophagy-related proteins beclin1, Atg5, Atg7, and P62. The results revealed that sotetsuflavone was able to promote the levels of expression of beclin1, Atg5, and Atg7, and induced a decrease in the levels of expression of P62 in A549 non-small cell lung cancer cells (**Figure 3**). Our results further indicated that the sotetsuflavone treatment group was rich in the levels of AVO compared to levels in the control group based upon observations resultant from orange staining (**Figure 3**). In order to further test the degree of changes in autophagy of NSCLC cells that were induced by sotetsuflavone, we observed the appearance of double-membrane autophagosomes by the use of transmission electron microscopy. Compared with the results from the control group, there was the obvious presence of autophagosomes or autophagic lysosomes which had accumulated in the sotetsuflavone treatment group (**Figure 3**). In conclusion, these results demonstrated that sotetsuflavone was able to induce autophagy in NSCLC A549 cells.

### Inhibition of Autophagy Promotes Apoptosis and Growth Inhibition of NSCLC Cells Induced by Sotetsuflavone

**Figure 4:**
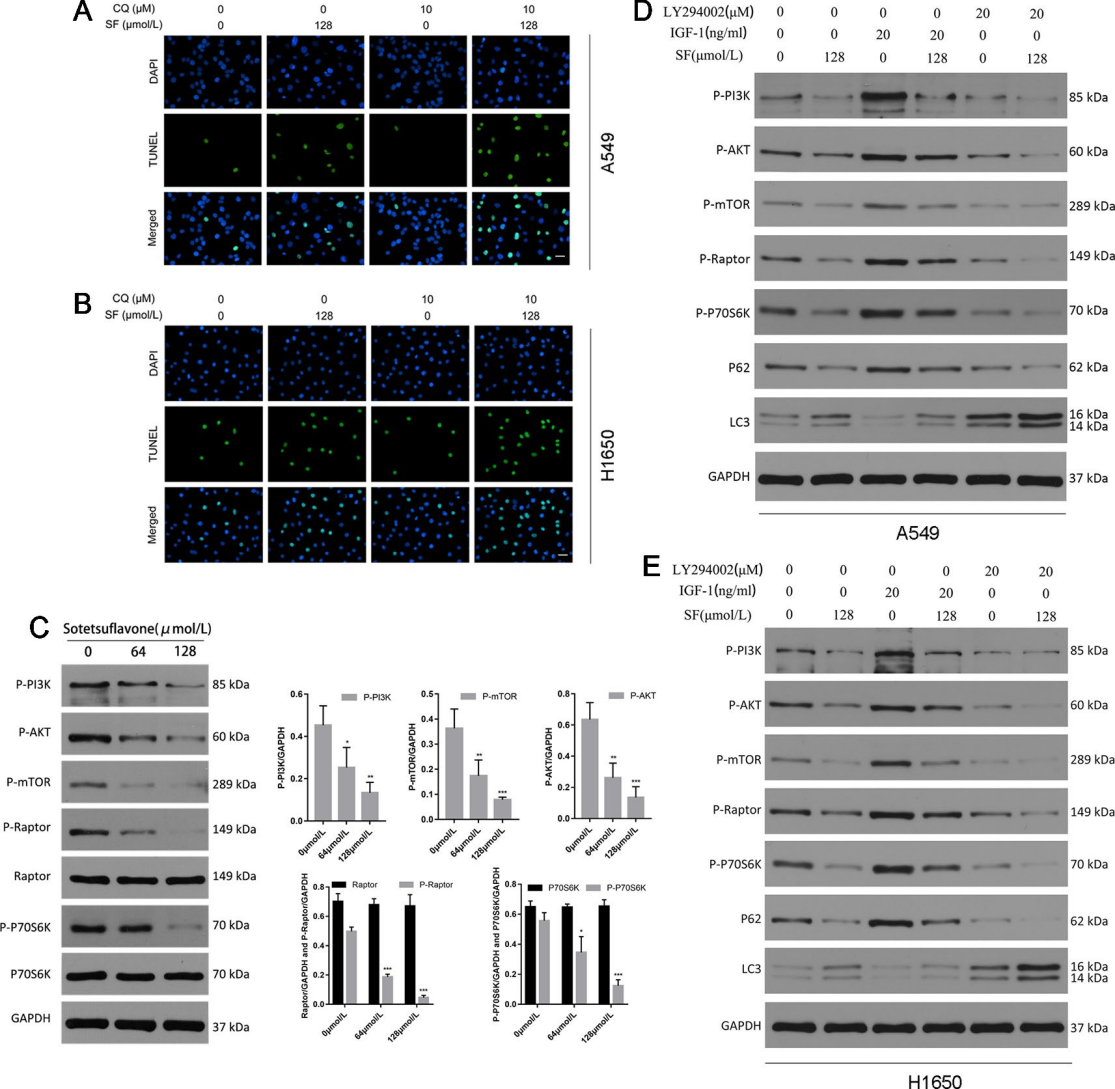
Inhibition of autophagy promotes apoptosis of non-small cell lung cancer cells induced by sotetsuflavone. Sotetsuflavone affects autophagy of non-small cell lung cancer cells by inhibiting the PI3K/AKT/mTOR signaling pathway. **(A)** and **(B)** A549 and H1650 cells were treated with or without sotetsuflavone (128 μmol/L) in combination with CQ (10 μM) for 24 h, then a TUNEL assay was used to detect apoptotic cells. **(C)** The levels of expression of p-PI3K, p-Akt, p-mTOR, Raptor, p-Raptor, p70S6K, and p-p70S6K in A549 cells were evaluated by Western blotting. GAPDH was used as a loading control. Gray scale analysis was performed to determine relative proportions of p-PI3K, p-Akt, p-mTOR, p-Raptor, Raptor, p-P70S6K, and P70S6K (*p < 0.05, **p < 0.01, ***p < 0.001 vs. control). A549 cells **(D)** and H1650 cells **(E)** were treated with or without sotetsuflavone (128 μmol/L) in combination with IGF-1 (20 ng/ml) or LY294002 (20 μM) for 24 h. Phosphorylated PI3K(p-PI3K), phosphorylated Akt(p-Akt), phosphorylated mTOR(p-mTOR), phosphorylated Raptor(p-Raptor), phosphorylated p70S6K(p-p70S6K), P62, LC3-1, and LC3-II were detected by Western blotting.

Because the relationship between apoptosis and autophagy is complex, in order to elucidate cross-talk between autophagy and apoptosis that was induced by sotetsuflavone in NSCLC cells, we then used autophagy inhibitors (CQ) to block autophagy, and then determined the effect of sotetsuflavone on apoptosis. TUNEL staining of the cells that were treated with sotetsuflavone was significantly enhanced in the presence of CQ (**Figures 4A, B**). Furthermore, we observed whether or not the anti-tumor effects in NSCLC A549 cells induced through sotetsuflavone could be enhanced by autophagy inhibitors. Our results indicated that the combination of CQ or LY294002 with sotetsuflavone was able to strengthen the inhibitory effect on A549 cells (**Figure S2**). In conclusion, the results indicated that autophagy induced by sotetsuflavone has a cytoprotective effect on apoptosis in NSCLC A549 cells. At the same time, our data also suggested that the inhibition of autophagy was able to enhance sotetsuflavone mediated anti-proliferation activities.

### Sotetsuflavone Affects Upon Autophagy of A549 Cells Through Inhibition of the PI3K/Akt/mTOR Signaling Pathway

The Akt/mTOR pathway is the main negative oriented regulation pathway of autophagy (Heras-Sandoval et al., 2014). Thus, we next explored whether or not the PI3K/Akt/mTOR pathway was inhibited in NSCLC A549 cells that had been treated with sotetsuflavone. Firstly, we used Western blotting in order to detect the levels of expression of phosphorylated PI3K, Akt, mTOR, Raptor, and p70S6K (a characteristic target of the mTOR1 complex). The degree of inhibition of the PI3K/Akt/mTOR pathway was mediated by the application of sotetsuflavone in NSCLC A549 cells which we confirmed by way of reducing the level and degree of phosphorylation of PI3K, Akt, mTOR, Raptor, and p70S6K (**Figure 4**). In addition, we further used activators and inhibitors of the PI3K/Akt signaling pathway, IGF-1 and LY294002, in order to determine whether or not sotetsuflavone was able to inhibit the PI3K/Akt signaling pathway. The results clarified that inhibitory effects of sotetsuflavone on the PI3K/Akt signaling pathway were reversed by application of a treatment of 20 ng/ml of IGF-1. In contrast, the combination of both sotetsuflavone and LY294002 was found to have significantly increased the degree of LC3-II conversion in non-small cell lung cancer A549 cells (**Figure 4**). We further verified this result for examinations of H1650 cells (**Figure 4**). These results demonstrated with a high degree of certainty that sotetsuflavone-induced autophagy was related to the inactivation of the PI3K/Akt/mTOR pathway in NSCLC cells.

### Sotetsuflavone Could Interact With Protein PI3K, Akt, and mTOR

**Figure 5:**
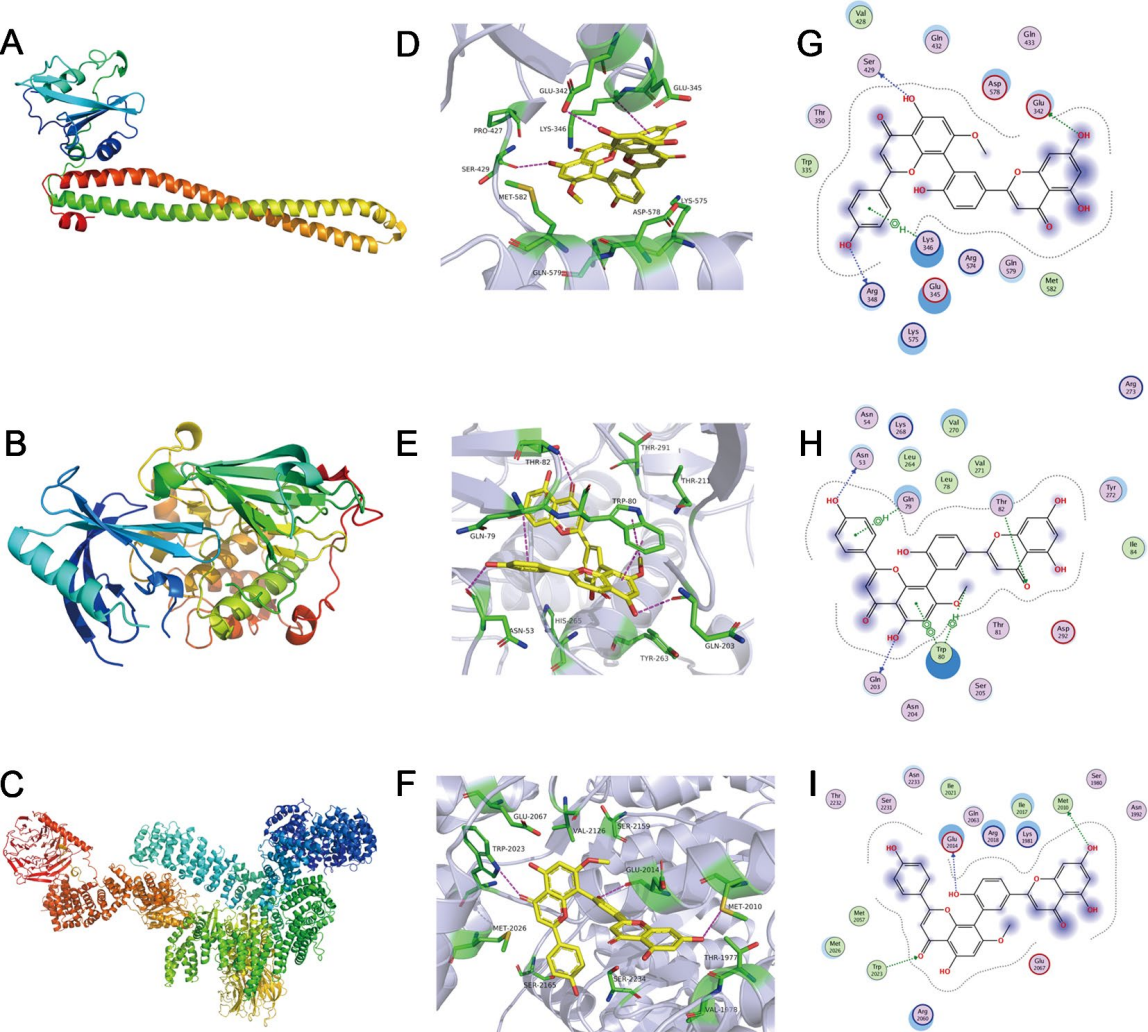
Sotetsuflavone could interact with proteins including PI3K, Akt, and mTOR. **(A)** 3D structures of human proteins PI3K (PDB-ID:5XGI). **(B)** The 3D structures of human proteins AKT(PDB-ID:4EGN2). **(C)** The 3D structures of human proteins mTOR (PDB-ID:6BCX3). **(D)** The 3D binding mode of sotetsuflavone with PI3K(PDB-ID:5XGI). **(E)** The 3D binding mode of sotetsuflavone with AKT(PDB-ID:4EGN2). **(F)** The 3D binding mode of sotetsuflavone with mTOR (PDB-ID:6BCX3). **(G)** The 2D binding mode of sotetsuflavone with PI3K(PDB-ID:5XGI). **(H)** The 2D binding mode of sotetsuflavone with AKT(PDB-ID:4EGN2). **(I)** The 2D binding mode of sotetsuflavone with mTOR (PDB-ID:6BCX3). In the image, sotetsuflavone is colored in yellow, and the surrounding residues in the binding pockets are colored in green. The backbone of the receptor is depicted as light blue cartoon.

The method of molecular docking is based upon computer analysis that is able to predict the binding affinity of a new chemical entity or drug according to its respective chemical structure. Molecular docking uses the application of mathematics, biology, and computer modeling in order to predict the affinity of small molecules for a specific receptor (Gupta et al., 2018). To investigate the binding mode of sotetsuflavone with human proteins PI3K, Akt, and mTOR, we carried out docking simulation studies. Our results indicated that the docking scores of sotetsuflavone were -7.97 kcal/mol, -7.18 kcal/mol, and -7.03 kcal/mol for PI3K, Akt, and mTOR, respectively. In summation, we found that sotetsuflavone was able to interact with Glu342, Ser429, and Lys346 of PI3K through hydrogen bond and π-hydrogen bond interactions (**Figures 5D, G**). Sotetsuflavone was found to interact with Asn53, Thr82, Gln203, Gln79, and Trp80 of Akt through hydrogen bonds, π-hydrogen bonds, and π-π bond interactions (**Figures 5E, H**). Sotetsuflavone was found to interact with Trp2023, Glu2014, and Met2010 of mTOR through hydrogen bond interactions (**Figures 5F, I**). The differences in binding models of sotetsuflavone and proteins lead to and resulted in different binding abilities. The computational results indicated that sotetsuflavone was able to interact with PI3K, Akt, and mTOR.

### Xenograft Tumor Growth Was Inhibited by Sotetsuflavone

**Figure 6:**
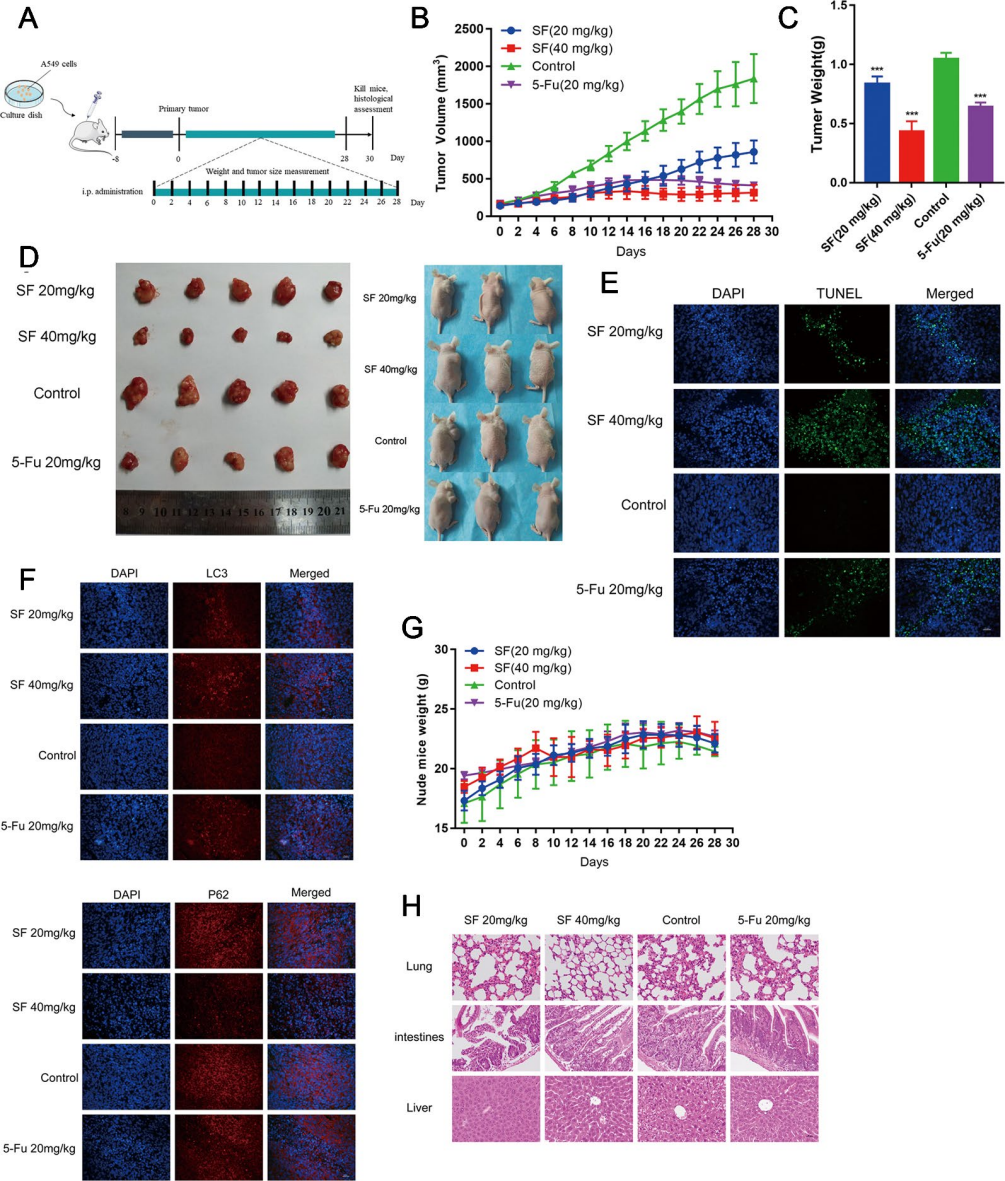
Sotetsuflavone inhibits the growth of xenograft mice bearing A549 tumors *in vivo*. **(A)** Establishment of xenograft mice bearing A549 tumors and the drug administration method. **(B)** Tumor volumes were measured and calculated every 2 days. **(C)** Tumor weight in the control group, 5-Fu (20 mg/kg), sotetsuflavone treatment groups (20 mg/kg, 40 mg/kg) (***p< 0.001 vs. control). **(D)** A549 cells were subcutaneously inoculated into the back of nude mice. The figure at the right side of the image showed the representative size of the four groups of tumors. **(E)** TUNEL staining (green) in tumor tissues; DNA was stained by DAPI (blue). **(F)** Representative images showed immunofluorescence staining of LC3 and P62. **(G)** The weight of nude mice was recorded every 2 days. **(H)** H&E stained the tumors, lungs, intestines, and livers of mice from each group, whereby the pathological changes of mice were observed, and the toxicity of sotetsuflavone was evaluated. Scale bars, 50 µm.

In order to further prove whether or not sotetsuflavone was able to exert the same anti-tumor effects *in vivo*, we evaluated such effects of sotetsuflavone *in vivo* by xenograft mice bearing A549 tumors. As shown in **Figure 6**, mouse modeling and drug administration were conducted. The results indicated that the applications in sotetsuflavone administration group were able to significantly reduce tumor volume (**Figure 6**). And, the results from tumor resectioning indicated that tumors treated with sotetsuflavone were much smaller than tumors were observed to have been in the control group (**Figure 6**). The mean tumor weight of the sotetsuflavone group was obviously lower than was the mean tumor weight in the control group (**Figure 6**). In addition, we detected levels of apoptotic cells in tumor tissues by using TUNEL staining, and the results clarified that there was sotetsuflavone induced tumor tissue damage (**Figure 6**). We also monitored the degree of toxicity of sotetsuflavone throughout the *in vivo* experiment. The treatment with sotetsuflavone was not found to have caused any abnormalities after a period of 28 days, and the H&E staining showed no serious morphological changes in organs of sotetsuflavone treated mice (**Figures 6G, H**). In order to confirm the evidence suggesting that autophagy formation was induced by sotetsuflavone, we further detected the levels of expression of LC3 and P62 in tumor tissues by using immunofluorescence assays. As shown in **Figure 6**, results indicated that sotetsuflavone was able to increase the levels of LC3 expression and decrease the levels of P62 expression. In conclusion, our results suggested that sotetsuflavone had a low toxicity *in vivo* and was still able to inhibit the growth of xenograft mice bearing A549 tumor.

## Discussion

Flavonoids are types of polyphenolic compounds with more than 4000 variations, and are widely found in various natural plants (Panche et al., 2016; Wang et al., 2019). There is a growing base of evidence that indicates that flavonoids or flavonoid derivatives have and can play a pivotal role in the dynamics of chemoprophylaxis and chemotherapy of types of cancer. Correspondingly, a high intake of flavonoids may be associated with reduced cancer risk, supporting the evidence for the protective effect of flavonoids against the onset and progression of cancer (Cao et al., 2011; Luo et al., 2015). Autophagy and apoptosis are strictly regulated processes of cell and tissue homeostasis, development, and disease (Doherty and Baehrecke, 2018). Recent studies have found and suggested that many anti-cancer drugs can cause autophagy in cancer cells. However, the role of autophagy in cancer treatment remains controversial (Maycotte et al., 2012; Galluzzi et al., 2017). Currently, there are four different functional forms that have been identified and have been shown to be involved in the treatment of cancer, including cytoprotective, non-protective, cytotoxic, and cytostatic autophagy (Sun et al., 2018). Autophagy is a mechanism for which the delivery of cell materials to lysosomes results in degradation, which ultimately leads to the basic renewal of cell components and provides energy and macromolecular precursors. Autophagy plays a crucial role in cell physiology and human diseases (Jmm et al., 2017). Thus, in our study, we sought to examine whether or not sotetsuflavone was able to induce autophagy in NSCLC cells, and evaluated potential mechanisms behind the effects of sotesufllavone.

Inhibiting the proliferation of tumor cells is a critical aspect with obvious importance for approaches to treat cancer (Wang et al., 2018b). Therefore, we examined the proliferation in A549 and H1650 cells by using both EDU and plate colony formation experiments. The results indicated that sotetsuflavone was able to significantly inhibit the proliferation of A549 and H1650 cells (**Figures 1B, C, F, G**). Concurrently, we found that the application of 0-160 µmol/L of sotetsuflavone had no significant toxic effects on BEAS-2B cells (**Figure 1**). These results further suggested that sotetsuflavone was able to specifically inhibit the proliferation of NSCLC cells. Apoptosis is a type of programmed cell death, which involves early destruction of cytoskeletal components, the preservation of organelles until the later stages of the process, and has been found to be caspase-dependent (Hsu et al., 2009). Thus, we next used TUNEL staining experiments in order to detect whether or not sotetsuflavone was able to induce apoptosis in A549 and H1650 cells. Results indicated that sotetsuflavone was able to cause apoptosis of A549 and H1650 cells (**Figure 2C**). Although we observed that the levels of both CDK1 and CDK4 were decreased in sotetsuflavone treated cells (**Figure 2E**), these can also be simply degraded by proteases, which are typically activated during apoptosis. Thus, cell cycle arrest may result from the degradation of CDKs, however, there is no evidence to suggest that sotetsuflavone can causes inhibition of cell proliferation directly, and this needs further clarification. Autophagy is another process and aspect of cell death that differs from apoptosis (Tsai et al., 2015). Autophagy plays an important role in tumor development and is considered to be an important mechanism in processes related to cell death. Many autophagy-related genes (ATG) are involved in the regulation of autophagy, such as including Beclin1, P62/SQSTM, LC3, and others that have been well described in the literature (Panda et al., 2015). Thus, to determine whether or not sotetsuflavone is able to induce autophagy in NSCLC cells, we examined the levels of expression of autophagy markers LC3-I/LC3-II and P62. The results indicated that sotetsuflavone was able to induce autophagy in NSCLC cells (**Figures 3** and **S1**). In addition, we clarified that the sotetsuflavone treatment group had results that suggested there was promotion of the levels of expression of beclin1, Atg5, and Atg7, and induced a decrease in the levels of expression of P62 (**Figure 3**). Furthermore, the formation of acidic vesicle organelles (AVO) is another important feature of autophagy (Sun et al., 2018). Thus, we tested this by using acridine orange staining. As predicted by the results from acridine orange staining, there was a greater abundance of AVO in the sotetsuflavone-treated group than was observed in the control group (**Figure 3**). In the detection of autophagosomes, Transmission electron microscopy is considered to be the gold standard for detection of the levels of autophagy and can qualitatively assess a series of ultrastructures (such as isolators, autophagic vacuoles, autophagosomes) that occur in autophagy (Yang et al., 2011). Our results indicated that there was an obvious accumulation of autophagosomes or autolysosomes in the results for the sotetsuflavone treatment group (**Figure 3**). Induction of autophagy in tumor cells has been found to be associated with inhibition of the PI3K/AKT/mTOR signaling pathway and such a result has been demonstrated in other types of tumors (Tsai et al., 2015; Wang et al., 2017). Additional research has indicated that autophagy induced by sotetsuflavone has a cytoprotective effect on apoptosis (**Figures 4A, B**). Furthermore, the application of sotetsuflavone was found to have induced autophagy and such a result may be achieved by way reducing the levels of expression of phosphorylation-PI3K, Akt, mTOR, and p70S6K in order to inhibit the PI3K/Akt/mTOR pathway (**Figure 4**). We therefore then treated the cells with IGF-1 (20 ng/ml) and sotetsuflavone (128 µmol/L), and found results that indicated that the inhibition of PI3K/Akt signaling by sotetsuflavone was reversed. In contrast, sotetsuflavone (128 µmol/L) used in combination with LY294002 (20 µM) significantly increased LC3-II conversion in non-small cell lung cancer cells (**Figures 4D, E**). These results fully support that autophagy induced by sotetsuflavone was associated with the inactivation of the PI3K/Akt/mTOR pathway in NSCLC cells. Molecular docking experiments are one of the most commonly used methods in for structure-based drug design. It also has been found to play an important role in the prediction of surface functional sites of protein molecules, protein ligand docking, and so forth (Leonardo et al., 2015). Molecular docking experiments further demonstrated that sotetsuflavone was able to interact effectively and efficiently with PI3K, Akt, and mTOR (**Figure 5**). We also demonstrated the anti-cancer effect of sotetsuflavone by use of xenograft mice bearing A549 tumors *in vivo*. Tumor volume (**Figure 6**) and tumor weight (**Figure 6**) decreased after treatment with sotetsuflavone, but body weight (**Figure 6**) was not significantly affected. The absorption and excretion of drugs in the body has the effect of inducing the phenomenon of enterohepatic circulation. Therefore, we used the HE staining method in order to detect pathological changes of intestine, lung, and liver tissues, and results indicated that sotetsuflavone was relatively non-toxic for the experimental animals (**Figure 6**). Meanwhile, we further tested and examined the effects of sotetsuflavone on apoptosis and autophagy in mouse tumor tissues (**Figures 6E, F**), and results indicated substantially consistent results with *in vitro* experiments.

## Conclusion

**Figure 7:**
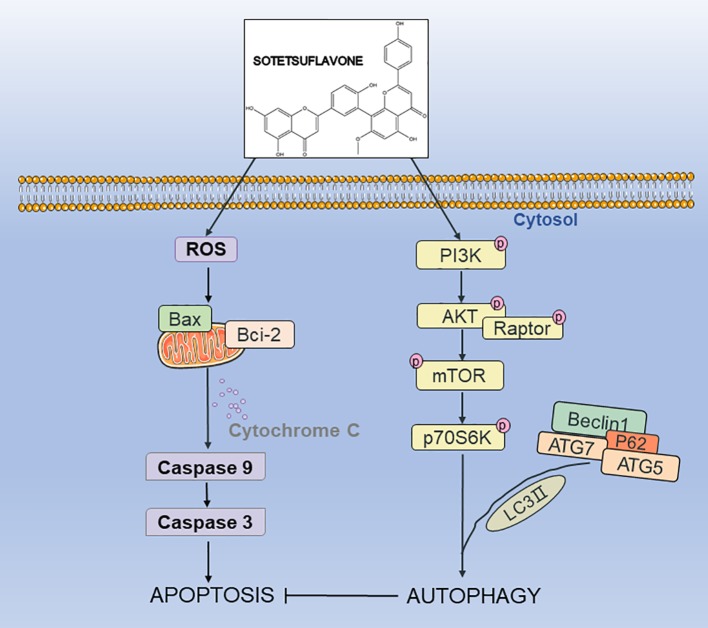
The possible mechanism of sotetsuflavone inhibited progression of non-small cell lung cancer. Sotetsuflavone could induce autophagy, apoptosis, and G0/G1 phase arrest, leading to the death of non-small-cell lung cancer cells.

In summary, our results indicated for the first time that sotetsuflavone had a strong anti-NSCLC effect both *in vitro* and *in vivo*. Sotetsuflavone was able to induce autophagy, and the effect was mediated by inducing excessive autophagy in NSCLC cells, thereby accelerating cell death. Our results strongly suggest that sotetsuflavone induced autophagy was associated with inactivation of the PI3K/Akt/mTOR pathway in NSCLC cells (**Figure 7**). Our study provides insight into the molecular mechanism by which sotetsuflavone induces cell death in NSCLC, which may help sotetsuflavone to become a drug for the treatment of anti-non-small cell lung cancer.

## Materials and Methods

### Materials, Reagents, and Antibodies

3-(4, 5-dimethylthiazol-2-yl)-2, 5-diphenyltetrazolium bromide (MTT) assay kit and HRP-Goat anti Rabbit secondary antibody were obtained from Sigma (St. Louis, USA). A549 cells were purchased from the Cell Resource Center of Peking Union Medical College (Beijing, China). H1650 cells were purchased from iCell Bioscience, Inc. (Shanghai, China). Sotetsuflavone was purchased from Must Biological Technology Co. Ltd. (Chengdu, China), and the purity of sotetsuflavone was determined to exceed 98% by use of HPLC. (**Figure 1**). Dulbecco’s modified eagle medium (DMEM) was bought from HyClone (Los Angeles, USA). Transwell plate was bought from Corning (New York, USA). Z-VAD, LY294002, and chloroquine (CQ) were bought from Selleck (Houston, USA). Human IGF-1 was purchased from Peprotech (New Jersey, USA). Anti-GAPDH, anti-CDK4, anti-cytochrome c, anti-cleaved caspase3, anti-Bcl-2, anti-P-PI3K(Y607), anti-P-P70S6K(T389), anti-P70S6K, and anti-P62 were all purchased from Abcam (Shanghai, China). Anti-cyclin D1, anti-Bax, anti-P-AKT(S473), anti-P-mTOR(ser2448), and anti-LC3 were all bought from CST (Cell Signaling Technology, Boston, MA, USA). Anti-Cleaved caspase9, anti-P-Raptor(ser792), and anti-Raptor were all bought from Affbiotech (Affinity Biosciences, Inc., USA). Invitrogen™ Trizol Reagent, PrimeScript™ RT reagent Kit with gDNA Eraser, and SYBR^®^ Premix Ex Taq™ were bought from Takara Biomedical Technology (Beijing) Co., Ltd. (Beijing, China). ELISA detection microplate reader (DR-200Bs) was bought from Wuxi Hiwell Diatek Instruments Co., Ltd. (Wuxi, China). StepOne™ Real-Time PCR System components were bought from Life technologies (California, USA). All other chemicals were determined to be of high purity and were purchased from commercial sources.

### Cell Culture and Drug Preparation

A549 cells (adenocarcinoma human alveolar basal epithelial cells) and H1650 cells (bronchoalveolar adenocarcinoma cell line) were cultured in DMEM medium containing 10% fetal bovine serum, penicillin and streptomycin at 100 U/ml, and in an atmosphere of 5% CO_2_ at 37°C. The sotetsuflavone solution was first prepared in dimethyl sulfoxide (DMSO) as a concentration of 200 mM, was then stored at −20°C, and then diluted to the desired concentration in DMEM medium before use in experiments (Wang et al., 2018b).

### Cell Viability and Colony Formation Assays

We used a MTT cell proliferation and cytotoxicity assay kit to detect the levels and degree of cell viability as previously described (Wang et al., 2018a). For the colony formation assay, cells were seeded at a density of 500 cells/well in 6-well plates and treated with different concentrations of sotetsuflavone. We changed the medium every 3 days, and after 14 days, we discarded the medium and washed the plates twice with PBS. Then, we added 1 ml of 100% methanol to each well, fixed the cells for 10 min, discarded the methanol, added 1 ml of 0.5% crystal violet solution to each well, and allowed staining to occur for 20 min. Finally, the number of cell colonies was counted, and the images were photographed. The level and degree of toxicity of sotetsuflavone upon normal lung epithelial cells (BEAS-2B) were detected according to and following all manufacturer protocols for applications of trypan blue staining.

### 5-Ethynyl-2′-Deoxyuridine (Edu) Proliferation Assay

Briefly, collected cells were incubated with 50 mM EDU and were then fixed, permeabilized, and stained with EDU according to and following all manufacturer protocols for the Cell-Light™ EdU Apollo^® ^643 In Vitro Imaging Kit (RiboBio). The nuclei were stained with DAPI at a concentration of 1 mg/ml for 20 min. Lastly, we determined the proportion of cells dosed with EDU.

### Wound-Healing Migration Assay and Transwell Invasion Assay

Cell migration and invasion abilities were assessed by wound-healing migration and transwell invasion assays as described previously (Wang et al., 2018b).

### Cell Cycle and Apoptosis Analysis

We used a cell cycle staining Kit (Sungene Biotech, Tianjin, China) to measure cell cycle. Briefly, after cells were treated with sotetsuflavone for 24 h, cells were then collected and washed twice with PBS. Then we gently dispersed and added 70% pre-cooled ethanol, and then fixed samples at 4°C overnight. We then resuspended samples in 500 µL of RNase/PI and stained samples for 20 min in the dark. We used both the Annexin V-FITC/PI apoptosis detection kit (Sungene Biotech, Tianjin, China) and the TUNEL(TdT-mediated dUTP nick end labeling)apoptosis detection kit (Roche, Shanghai, China) to detect cell apoptosis. Briefly, cells were treated as previously described, and the resultant collected cells were washed once with pre-cooled PBS. We then resuspended the cells in 300 µl of pre-cooled PBS diluted Binding Buffer, followed by the addition of Annexin V-FITC of 5 µl, then samples were incubated for 10 min in the dark. We next added PI in the amount of 5 µl in order to stain the cells followed by incubation for 5 min in the dark. Using BD FACS Calibur (BD Biosciences, Mountain View, CA, USA) we measured the levels and degree of cell apoptosis (Zhang et al., 2019a). For TUNEL analysis, briefly, cells were incubated with TUNEL labeling (marked with Green fluorescence) mix for 60 min at 37°C, and then we used DAPI (marked with Blue fluorescence, Sigma, USA) for double dyeing. The resultant sections were photographed using fluorescence based microscopy.

### Tem Detection

After the cells were treated with sotetsuflavone for 24 h, we then collected the cells and fixed them in 2.5% glutaraldehyde, followed by placing the cells at a temperature of 4°C overnight. We then rinsed the cells 3 times with PBS, and fixed the cells with 1% aqueous osmium for 2 h, followed by dehydration with 45%, 55%, 70%, 85%, 95%, 100% I, and 100% II of ethanol. We then impregnated, embedded, and polymerized cells with Epon 812 resin, using an ultra-thin microtome (Leica, Jena, Germany) to trim and slice (60-70 nm) the cells. Next, we dyed sampled by using 100 µL of aqueous uranyl acetate and lead citrate. Lastly, we used transmission electron microscopy (Hitachi HT7700, Tokyo, Japan) to observe the change of the cells during and after treatments.

### Acridine Orange Staining

Cells were treated with different doses of sotetsuflavone for 24 h. Then, cells were fixed by using 95% ethanol and stained with acridine orange working solution for 5 min at room temperature. We followed these steps by examining results through confocal microscopy (Zeiss, Oberkochen, Germany).

### Immunofluorescence Analysis

Cells were grown in a confocal dish and treated with varying concentrations of sotetsuflavone for 24 h. Next, cells were immobilized in paraformaldehyde for 10 min at room temperature, then we washed cells with ice-cold PBS and used 1% bovine serum albumin for blocking for a period of 1 h. The levels of expression of LC3 were determined by incubating the cells for 2 h at 4°C with the addition of the anti-LC3 primary antibody, next we incubated samples with the secondary antibody for 1 h. Then, we washed the cells three times with PBS, followed by staining with 0.1 µg/ml of DAPI in PBS for 10 min. We then again washed the cells with PBS. Finally, we used fluorescence based microscopy (Olympus, Tokyo, Japan) in order to observe the intensity of fluorescence.

### RNA Isolation and Quantitative Real-Time PCR

Isolation of RNA and target mRNA and their levels of expression were analyzed by use of quantitative real-time PCR assays which were performed as described previously (Wang et al., 2018b). The levels of expressed mRNA of GAPDH were used as an endogenous control. Primer sequences used for quantitative real-time PCR were as follows: For *Beclin1*, forward 5`-GACAGAG​CGATGGTAGTTCTGG-3` and reverse 5`-TGGGCTGTGGTA​AGTAATGGAG-3`; for *Atg5*, forward 5`-TGTTTATTCGTCGG​TTCATTTTG-3` and reverse 5`-CAGCTTAGTGTTCCCT​GCATTC-3`; for *Atg7*, forward 5`-TTCCTCCTCTTGACATTT​GCAG-3` and reverse 5`-TATCTTCGTCCTTTGACCTTG​G-3`; for *P62*, forward 5`-GATGAGGAAGATCGCCTTGG-3` and reverse 5`-CTTCGGATTCTGGCATCTGTAG-3`; and for *GAPDH*, forward 5`-CATCATCCCTGCCTCTACTGG-3` and reverse 5`-GTGGGTGTCGCTGTTGAAGTC-3`.

### Protein Extraction and Western Blot

Western blotting based detections were performed according to the previous descriptions from Wang et al. (2018a). Briefly, we extracted total cell proteins, and then lysed the samples in a RIPA buffer with an added protease inhibitor. The lysate was then separated by using sodium dodecyl sulfate–polyacrylamide (SDS-PAGE) gel electrophoresis gel, then we transferred the lysate to the polyvinylidene fluoride (PVDF) membranes, and used tris-buffered saline Tween-20 (TBST) in 5% skim milk as a blocking solution for 1 h. We then incubated samples with the primary antibody overnight at 4°C, and used the horseradish peroxidase-labeled secondary antibody to incubate the membranes. Lastly, we examined protein bands and visualized them by using an enhanced chemiluminescence kit (Millipore, Billerica, MA, USA).

### Molecular Docking

Molecular docking experimental analyses were conducted in MOE version 2018.0101 (2018). The 2D structures of sotetsuflavone were drawn by using ChemBioDraw 2014, and 3D structures in MOE were converted by energy minimization. The 3D structures of the PI3K protein (**Figure 5**), AKT protein (**Figure 5**), and mTOR protein (**Figure 5**) were downloaded from the RCSB Protein Data Bank. The PDB-ID were 5XGI, 4EJN (Ashwell et al., 2012), and 6BCX (Yang et al., 2017), respectively. Prior to docking, the force field of AMBER10: EHT and the implicit solvation model of the Reaction Field (R-field) were selected. MOE-Docking was used to perform molecular docking simulations of the sotetsuflavone with the corresponding proteins. The docking workflow followed the "induced fit" protocol, in which side chains of the receptor pocket were allowed to move according to ligand conformations, with a constraint upon their positions. The weight used for tethering side chain atoms to their original positions was 10. For each ligand, all docked poses were firstly ranked according to London dG scoring. Then, a force field refinement was carried out on the top 20 poses followed by a rescoring of GBVI/WSA dG. The resultant conformations with the lowest free energies of binding were selected as the best (probable) binding modes. Molecular graphics were generated by using PyMOL (Song et al., 2016; Guo et al., 2019).

### Animal Studies

All animal procedures in this investigation were approved and conformed to the Guide for the Care and Use of Laboratory Animals published by the US National Institutes of Health (NIH publication No. 85-23, revised 1996). For xenograft studies, male BALB/C nude mice aged 4 weeks were bought from Beijing Weitong Lihua Experimental Animal Co., Ltd. (Beijing, China), and were maintained under supervision and in pathogen-free conditions. A549 cells (concentration of 1×10^7^) in PBS were injected subcutaneously to the right side of the back of each nude mouse, and the mice showed palpable tumors. After tumors formed in nude mice, the mice were divided into 4 groups (each group, n = 5) in a random manner. The sotetsuflavone treatment group had concentrations of 20 and 40 mg/kg, and the model control group and the 5-Fu administration group had concentrations of 20 mg/kg which were administered by intraperitoneal injections every 4 days for 4 weeks. DMSO was used as a control. Measurements of tumor size and body weights in nude mice were made every 2 days from the initial injection. We made measurements of tumor size by using a vernier caliper, and we calculated tumor volume by using the formula: V = L × W^2^ × 0.52 (L, long axis; W, short axis). When the experiment concluded, nude mice were euthanized, and then tumor tissues were separated and photographed immediately. The weight of each nude mouse tumor was determined. The nude mice took subcutaneous tumor tissue, lung, intestine, and liver tissue samples, and some of these samples were fixed in 4% paraformaldehyde while another part of each respective sample was frozen in liquid nitrogen and placed in long-term storage at -80°C. For histopathological analysis, briefly, tumor tissues were collected, fixed with formalin buffer, and embedded in paraffin. Hematoxylin and eosin (H&E) were performed as previously reported. We then used a TUNEL apoptosis detection kit (Roche, Shanghai, China) to measure cell apoptosis. The levels of expression of LC3 and P62 in mouse tumor tissues were detected by using immunofluorescence analyses, and the details of the detection method are as described above (Zhang et al., 2019b).

### Statistical Analysis

We used IBM SPSS 20.0 statistical software to analyze experimental data. The experiments were repeated at least three times. Measurement data conformed to normal distributions and were expressed as mean ± standard deviation (SD). For comparisons of means between groups, we used one-way ANOVA analysis. When the variance was observed to homogeneous, the LSD and SNK methods were used, and when the variance was observed to be uneven, we used the Dunnett T3 method. A level of P < 0.05 was considered to be statistically significant.

## Data Availability Statement

All datasets generated for this study are included in the article/**Supplementary Material**.

## Ethics Statement

The animal study was reviewed and approved by Biological and Medical Ethics Committee, Minzu University of China.

## Author Contributions

SW is responsible for experimental design, data acquisition and analysis, and writing of manuscripts. XX and TLe obtained funding, technical support, proof-reading, and revision of the manuscript. YH technical support and interpretation of data. TLi receives funding, experimental design, learning supervision, and manuscript revision.

## Funding

The study was financially supported by the grants from the autonomous foundation of Key Laboratory of Ethnomedicine (Minzu University of China), Ministry of Education (Grant No. KLEM-ZZ201902), the National Natural Science Foundation of China Grants (Grant No. 81973977, 81802995), Zhejiang province public welfare funds (Grant No. 2017C33092, LGF19H280004).

## Conflict of Interest

The authors declare that the research was conducted in the absence of any commercial or financial relationships that could be construed as a potential conflict of interest.
