# Supplementary material for: Sotetsuflavone Induces Autophagy in Non-Small Cell Lung Cancer Through Blocking PI3K/Akt/mTOR Signaling Pathway in Vivo and in Vitro
Source: Front Pharmacol. 2019 Dec 5;10:1460. doi: 10.3389/fphar.2019.01460 (PMC6915081; doi:10.3389/fphar.2019.01460)
Supplement: Supplementary file 1 [file DataSheet_1.docx]

**Supplementary materials**
**For**
**Sotetsuflavone Induces Autophagy in Non-small Cell Lung Cancer Cells through Blocking PI3K/Akt/mTOR Signaling Pathway in Vivo and in Vitro**

Shaohui Wang^1,2,4*^, Xiaoling Xu^3^, Yanlan Hu^1,4^, Tao Lei^3^, and Tongxiang Liu^1,4*^

1 Key Laboratory of Ethnomedicine (Minzu University of China), Minority of

Education, Beijing 100081, P.R. China.

2 Medical College, Qingdao Binhai University, Qingdao, Shandong 266555, P.R. China.

3 Department of Medical Oncology, Institute of Cancer Research and Basic Medical Sciences of Chinese Academy of Sciences, Cancer Hospital of University of Chinese Academy of Sciences, Zhejiang Cancer Hospital, Hangzhou, Zhejiang 310022, P.R. China.

4 School of Pharmacy, Minzu University of China, Beijing 100081, People’s Republic of China

***Corresponding Author:** Tongxiang Liu, E-mail: tongxliu123@hotmail.com.

Shaohui Wang, E-mail: winter9091@163.com.

**Figure Legend for Supplementary Data**

**Supplementary Figure 1.** Sotetsuflavone activate autophagy flux in NSCLC A549 cells.

**Supplementary Figure 2.** Inhibitors CQ and LY294002 can enhance the inhibition of sotetsuflavone on the proliferation of non-small cell lung cancer A549 cells.


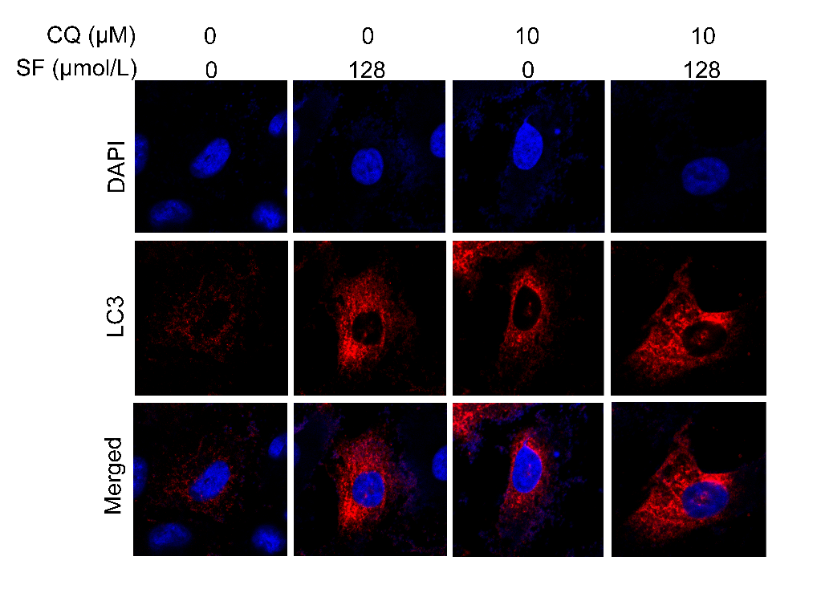


**FIGURE S1** Sotetsuflavone activate autophagy flux in NSCLC A549 cells. A549 cells were treated with 128 μmol/L sotetsuflavone and with or without 10 μM CQ for 24 hours. LC3 spots and nuclei were observed under a confocal microscope. Scale bar: 5μm.


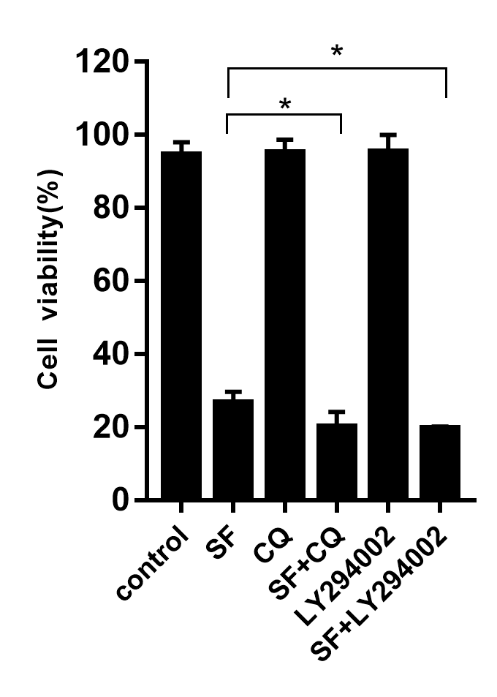


**FIGURE S2** Inhibitors CQ and LY294002 can enhance the inhibition of sotetsuflavone on the proliferation of non-small cell lung cancer A549 cells. A549 cells were treated with 128 μmol/L sotetsuflavone and with or without 10 μM CQ or 20 μM LY294002 respectively for 24 hours. And the cell viability was measured by MTT assay.
